# When Optimal Feedback Control Is Not Enough: Feedforward Strategies Are Required for Optimal Control with Active Sensing

**DOI:** 10.1371/journal.pcbi.1005190

**Published:** 2016-12-14

**Authors:** Sang-Hoon Yeo, David W. Franklin, Daniel M. Wolpert

**Affiliations:** 1 Computational and Biological Learning Lab, Department of Engineering, University of Cambridge, Cambridge, United Kingdom; 2 School of Sport, Exercise and Rehabilitation Sciences, University of Birmingham, Edgbaston, Birmingham, United Kingdom; 3 Department of Sport and Health Sciences, Technical University of Munich, Munich, Germany; Northeastern University, UNITED STATES

## Abstract

Movement planning is thought to be primarily determined by motor costs such as inaccuracy and effort. Solving for the optimal plan that minimizes these costs typically leads to specifying a time-varying feedback controller which both generates the movement and can optimally correct for errors that arise within a movement. However, the quality of the sensory feedback during a movement can depend substantially on the generated movement. We show that by incorporating such state-dependent sensory feedback, the optimal solution incorporates active sensing and is no longer a pure feedback process but includes a significant feedforward component. To examine whether people take into account such state-dependency in sensory feedback we asked people to make movements in which we controlled the reliability of sensory feedback. We made the visibility of the hand state-dependent, such that the visibility was proportional to the component of hand velocity in a particular direction. Subjects gradually adapted to such a sensory perturbation by making curved hand movements. In particular, they appeared to control the late visibility of the movement matching predictions of the optimal controller with state-dependent sensory noise. Our results show that trajectory planning is not only sensitive to motor costs but takes sensory costs into account and argues for optimal control of movement in which feedforward commands can play a significant role.

## Author Summary

The dominant theory of how movements are planned suggests that a task specifies a motor cost in terms of effort and accuracy and the motor system chooses a movement to minimize this cost. The pre-eminent theory is that this results an optimal feedback controller that both generates the movement and can correct online for disturbances that arise due to factors such as noise. However, current formulations of the optimal feedback control problem have assumed that sensory information has noise independent of the action, or noise that simply scales with movement speed. In reality, visual and proprioceptive noise of limb position vary substantially with the state of the body. We show that including such variation in sensory feedback qualitatively changes the optimal solution so that feedback control alone is no longer optimal for the task and an additional feedforward controller must be used. To test this idea, we develop a novel motor learning paradigm in which we can vary the sensory noise on visual feedback of the limb and show that human participants adapt to such a perturbation to produce complex reach paths which are well accounted for by our model.

## Introduction

Despite the abundance in the degrees of freedom of the human body, our movements occupy a very small subset of all possible movements, showing remarkably stereotypical patterns of motion for a given task. There is a long history which posits that such stereotypy arises from each possible movement inducing a cost and the selected movement is the one that minimizes the cost [for reviews see 1,2]. For example, in optimal control the cost is defined as a combination of a number of sub-costs that are related to the quality of the movement, which are generally divided into costs for external goals (such as accuracy and precision) and regularization costs (smoothness or energy consumption). An optimal controller that minimizes the overall cost generally has a purely feedback form, called an optimal feedback controller, where the sensory information obtained during the execution is exploited online to determine the next motor output [3–5]. These time-varying feedback controllers both generate the movement and optimally correct for any variability that arises from sources such as noise. This concept of optimal feedback control has been successfully linked to the neural control of movement [for reviews see 2,6].

For optimal control and other planning models, with few exceptions [7,8], the focus has been on the cost of the movement per se, a highly motor-centric view of action. This stands in stark contrast to the field of active sensing [for a review see 9] in which actions are used to gather sensory information relevant for a given task. Optimal feedback control requires a state-estimation process to estimate the current state of the body, but the models have assumed that there is either no noise, or constant noise, on the sensory inputs. This is a crude approximation to the state-dependence of sensory variability known to occur in both the proprioceptive and visual localization of the limb [10–13].

Here we propose that planning of movement does not only consider how to make a movement based on the sensory information, but also has to take into account the consequences of the movement in shaping the upcoming sensory afferents, which will be used to guide the movement. As a result of these bi-directional considerations the control process could choose to make movements that are not optimal in terms of the accuracy or energy efficiency, in order to improve the quality of the sensory feedback. This may actually increase the regularization cost, but may reduce the cost for external goals by improving the accuracy and the precision of the movement due to the enhanced sensory information. Here we develop an optimal feedback control framework in which the cost is still comprised solely of accuracy and effort but because noise on the sensory input is state-dependent, it leads to a control policy which is a combination of a feedforward and feedback command. By experimentally manipulating the state-dependent noise, we provide evidence that humans are able to develop sensorimotor strategies to reduce the overall cost, and this behavior can be reproduced by our optimal control model.

## Results

Human participants grasped the handle of a robotic interface and were asked to make reaching movements between targets located in the horizontal plane. We used a monitor-mirror system that both prevented the subjects seeing their own arm and allowed us to project images into the plane of the movement [14]. The position of the hand was displayed online as a red cursor. To control state-dependent sensory noise we introduced a “velocity dependent visibility field”, where the visibility of the hand cursor (alpha value between 0, invisible, and 1, fully visible) depended on the hand velocity. Such velocity-based perturbations have typically been applied in terms of force fields which perturb a movement [15,16], whereas here there is no physical perturbation to the movement, but a variation in the ability to see the location of the hand during the movement based on the velocity, thereby affecting sensory noise.

### Experiment 1: Movements in a visibility field

**Fig 1:**
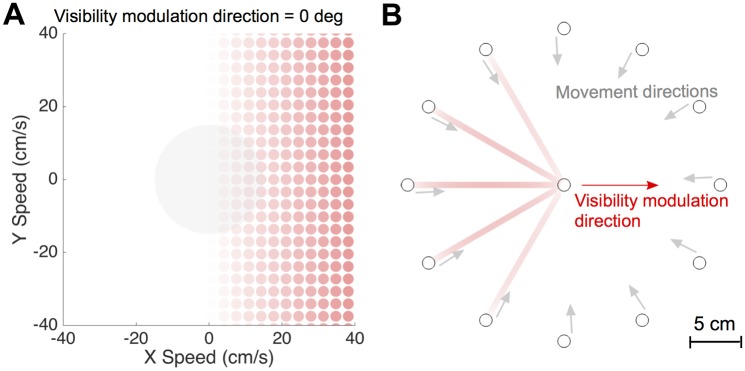
Experimental paradigm and visibility field. **(A)** Participants made reaching movement in the horizontal plane with visual feedback of their hand displayed as a red cursor (they could not see their arm or hand). We imposed a visibility field in which the alpha (transparency) of the red hand cursor depended on the hand velocity. In this example, the visibility modulation direction is 0° (rightward) and each circle show the appearance of the cursor for that hand velocity. The central grey circle shows the typical range of speed used in the experiment. **(B)** Cursor appearance for typical straight line movements to the central target for the visibility field shown in (A). Note that the actual experiments were performed on a black background.

The visibility (alpha level; as used in OpenGL) was proportional to the component of the speed in a given direction (the visibility modulation direction *θ*, see Materials and Methods for details). Therefore, for a visibility modulation direction of 0° (rightward), when stationary the hand cursor was invisible and increased in visibility with rightward motion but remained invisible for leftward motion (Fig 1A). Subjects performed center-in reaching movements from 12 starting points to a center target in the velocity-dependent visibility field. The visibility of the cursor for straight line movement to the central target is shown in Fig 1B, with some movement directions having variation in visibility while for others the cursor would be invisible throughout. To ensure that the task could not be solved based on proprioception alone, we included a small random translation offset between hand and cursor on each trial, thereby requiring the use of vision.

**Fig 2:**
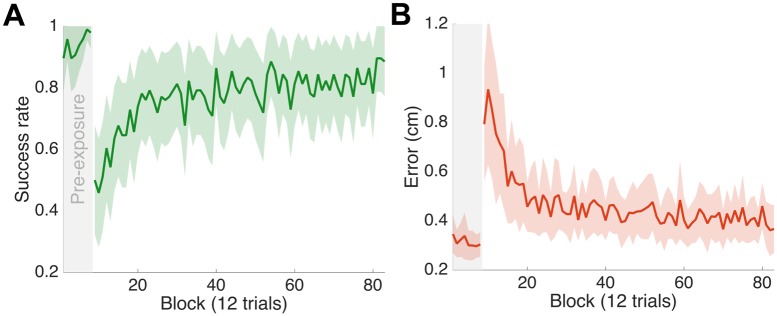
Performance in Experiment 1. **(A)** Success rate across blocks shown as mean ± s.e. across subjects. The success rate was the proportion of trials in which subjects placed the cursor within the target within 3 seconds of the target appearing. The first 8 blocks are pre-exposure trials. Each block consists of a movement from each starting location to the central target. **(B)** Error, the distance between the target and the final cursor position at the end of the trial, across blocks in the same format as (A).

There were significant changes in performance between pre-exposure, early and late exposure (F_2,6_ = 33.05; p = 0.01 for success rate and F_2,6_ = 28.125; p = 0.001 for final error). Prior to the introduction of the visibility field, when the cursor was fully visible at all times, subjects had both a high success rate (Fig 2A) and low final error (Fig 2B). On the introduction of the velocity-dependent visibility field, there was a large drop in performance (p<0.001 for both success and error; post hoc pairwise planned comparisons) followed by a significant increase in performance (p<0.001 for both success and error). The final performance at the end of the experiment was not significantly different from late pre-exposure (p = 0.051 for success and p = 0.086 for error).

**Fig 3:**
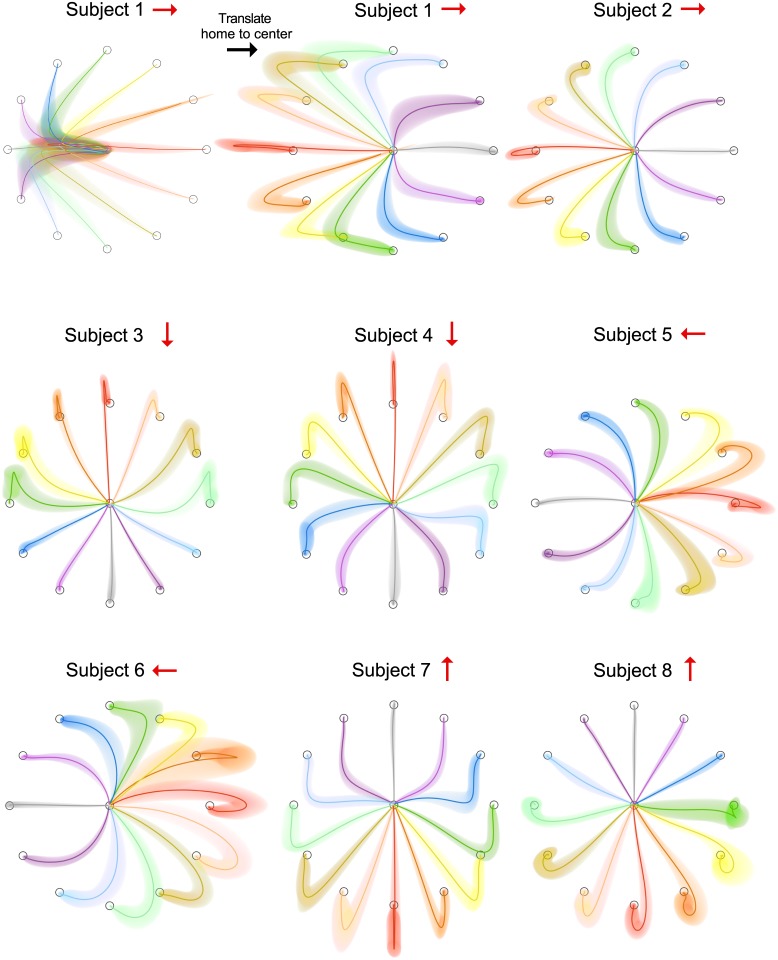
Hand paths in Experiment 1. Hand paths for each subject averaged over the final 10 blocks of the exposure phase (mean ± s.d.). Top left shows the actual hand paths for a typical subject. For clarity each hand path has then been translated to share the same starting location (top middle). For different subject the visibility modulation direction was chosen to be one of the 4 cardinal directions, shown by the red arrows.

Prior the perturbation, subjects made typical, straight-line movements to the target. However, the final 10 blocks of the exposure phase showed paths that were highly curved (Fig 3, top left panel). To better visualize the paths, for display purposes only, we transformed the original center-in movements to center-out movements by translating the start position of each movement to the center (original and transformed version shown for Subject 1 in Fig 3). All subjects showed curved movement paths, with the curvature dependent on the direction of the visibility modulation direction (Fig 3, red arrows). In general, movements in the visibility modulation direction were straight and became progressively curved away from this direction.

**Fig 4:**
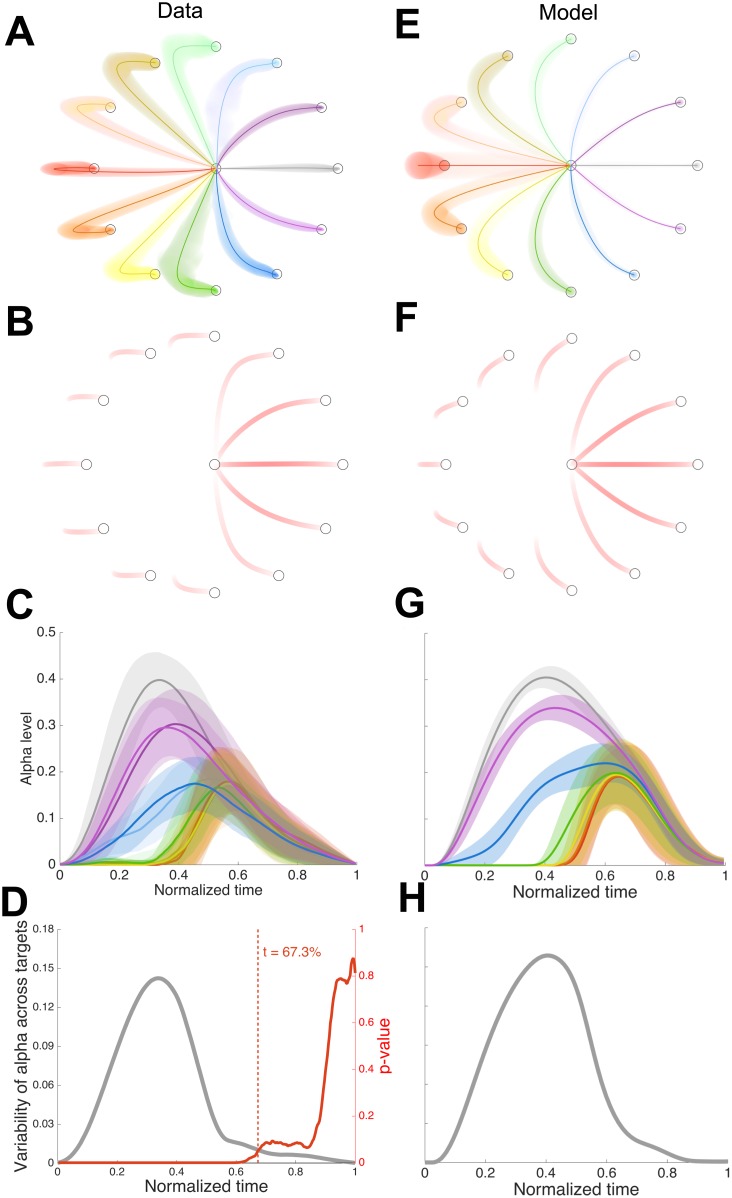
Empirical and simulated behavior in Experiment 1. **(A)** average hand paths across subjects (mean ± s.d. across the subjects). **(B)** cursor visibility corresponding to the average hand trajectories in (A). **(C)** Alpha level (visibility) against normalized time for each movement direction (mean ± s.d. across the subjects). Colors as in (A). **(D)** Variance of alpha across the 12 target directions. Red line shows the p-value for the data based on an ANOVA across the 12 directions for each normalized time. The vertical red dashed line shows the last time p<0.05. (**E**)-(**H**) same as (A)-(D) for the model.

To combine data across subjects we rotated all the trajectories for each subject to make the effective visibility modulation direction 0° (i.e. rightwards). The observed consistency of the trajectory patterns (Fig 4A) demonstrates that subjects adopted a similar sensorimotor strategy as a result of learning. First, the paths have at most a single point of inflection indicating that subjects made movements that switch between the invisible and the visible states at most once, rather than making more complex movements that switch the visibility states multiple times during the movement. Second, all subjects preferred to increase the visibility of the cursor at the end of the movement rather than at the beginning. That is for all movements (except the trials where the target direction was the same as the visibility modulation direction) subjects deviated away from the target in a direction that would produce a reduced initial visibility compared to the straight line movement to the target so as to approach the target in a direction that would increase visibility. When the target was located in the direction opposite to the visibility modulation direction (red trajectories), subjects chose to overshoot and return to the target so that the cursor became visible during the return.

This tendency to regulate late visibility is illustrated by plotting the cursor appearance along the paths (Fig 4B) and modulation of the alpha level with time (Fig 4C). Although there are large differences in the alpha levels across the 12 movement directions during the early and intermediate phases of the movement (variance across direction shown in Fig 4D, grey line), the last part of the movement tends to have a more similar pattern of alpha. This apparent regulation of the alpha level suggests that subjects may control their movement so that they could see a specific visual pattern of the cursor in the latter phase of the movement. To confirm this observation, we performed a sequential statistical test on the temporal profile of the alpha level and identified the time point after which the alpha levels of movements in the twelve different directions became indistinguishable from each other. For each time point, we performed a one-way ANOVA testing the main effect of movement direction (with subject as a random factor). The resultant time course of uncorrected p-value is shown in Fig 4D (red line) and there was no significant effect of the movement direction once two-thirds of the movement time had elapsed. This corresponds to ~337 ms before the cursor arrived at the target (average duration 1.012 s). Note that by not correcting the p-values we show that at least the last 337 ms of the movement has similar alpha values.

We developed an optimal feedback control model of our task that includes state-dependent sensory noise. This was based on a Linear Quadratic Gaussian formulation that includes the signal-dependent motor noise and state-dependent sensory noise. The formulation of the model and the resultant algorithm for finding the optimal controller and Kalman filter, through iterative updates, are based on previous work [17]. However, there is a key difference in that to model the state-dependence of the visual feedback we included uncertainty of the sensory information that had an affine form with respect to the state. That is we equate the reduction in hand cursor visibility to the increase in noise on hand position feedback. The implication of this affine characteristic is that sensory uncertainty changes in a dynamic state-dependent way. Unlike previous formulations in which any movement tends to increase sensory noise [17], in our formulation sensory noise depends both on the particular movement and can actually decrease for some movements. Therefore, the quality of sensory feedback depends on the action, allowing us to incorporate a form of active sensing within the optimal control framework. We derived the solution for this problem (see S1 Text) and unlike the problem without state-dependent noise on sensory input, the resultant controller now has a hybrid form containing both feedback and feedforward terms. We used this formulation to simulate the optimal movements under the visibility field. Results of the simulation (Fig 4E–4H) suggests that our optimal control model shares many of the features of the observed trajectories. The optimal solution is no longer to move directly to the target for most directions, but to move in directions in which sensory feedback is beneficial for the task. Comparing the observed and the predicted trajectories (Fig 4A and 4E), the model reproduces a similar pattern of the curvature as a function of target direction relative to visibility direction (*θ*) including the observed overshoot-return behavior when the movement was made in a direction opposite to *θ*.

**Fig 5:**
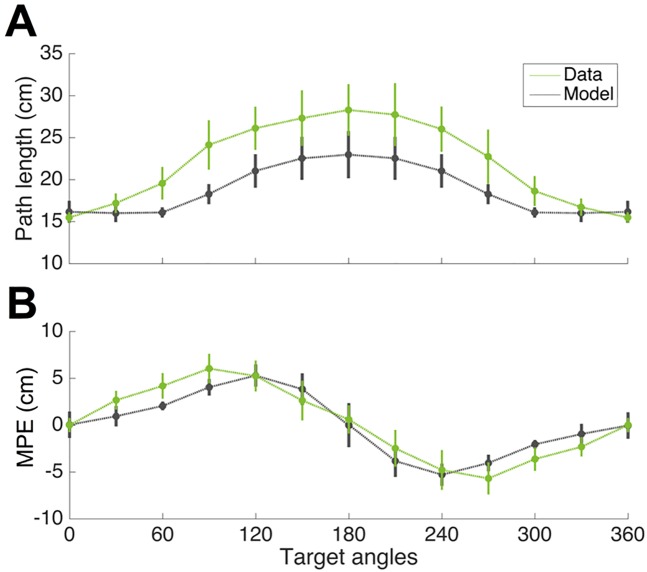
Kinematic attributes of empirical and simulated movements. **(A)** Path length as a function of movement direction for empirical (mean across subjects ± s.d.) and simulation (mean ± s.d. from Monte Carlo simulations). **(B)** As (A) but for MPE (Maximum Perpendicular Error).

To compare the trajectories, we calculated the (signed) deviation from a straight line movement to the target at 200 points along each path and correlated the model predictions with the mean trajectories which gave an R^2^ of 0.85. To further compare the similarity, we performed additional analyses on how the optimal control model predicts two common kinematic measures, path length and maximum perpendicular error (MPE). For trajectories in the last 10 blocks of trials, we calculated the signed MPE (positive when the cursor is deviated in a counter-clockwise direction from the straight line from home to target) and the path length for each movement direction. These were then compared with the ones predicted by our optimal control model, estimated by 3000 Monte Carlo simulations (as sensory and motor noise lead to variations in each simulation) for each direction. The result summarized in Fig 5 demonstrates that their overall patterns are qualitatively similar although the optimal paths are in general longer.

Simulated alpha level profiles in Fig 4G suggest that our optimal controller also shows the tendency to regulate the late visibility as it produces a tight overlap among alpha levels across the 12 movement directions after around two-third of the movement (Fig 4H). We performed a parameter sensitivity analysis to confirm that this tendency is not limited to a specific choice of model parameters (see Sensitivity analysis). Taken together, these observed similarities in the pattern of trajectory and the alpha level between data and the optimal control model suggest that the subjects were able to find a close to optimal strategy in the presence of a visibility field.

Notwithstanding these similarities, there also exist some differences between the observed and the predicted trajectory patterns. For example, when the cursor was approaching the target, subjects preferred to rapidly change their movement direction to the visibility modulation direction whereas the optimal control model predicts a rather smooth steering of the movement direction towards the visibility modulation direction over the entire duration of the movement (Fig 4A and 4E).

The optimal control model predicts that the motor command should be a combination of a feedback and a feedforward controller. Moreover, we show (see S1 Text) that the controller from our model can be decomposed into two separate controllers acting in orthogonal directions. Therefore, the component of hand motion orthogonal to the visibility modulation direction (*θ*) is controlled only by a feedback controller, whereas the component of motion in the direction of *θ* has both feedback and feedforward components. We can show the importance of the feedforward component by considering the movements purely orthogonal to *θ* (Fig 4A, up and down movements). For these, if the controllers were both purely feedback controllers, then the curved movements we observe, could not arise (as in this case only the orthogonal component generates commands leading to movements along a single axis, see S1 Text). Moreover, if we consider the component of movement that is orthogonal to *θ*, then it should be purely feedback with, for example, peak velocity scaling with target distance in that component and the peak velocity occurring at the same time independent of target direction. In contrast, the feedforward component along *θ* means that no such relation need exist in this direction.

**Fig 6:**
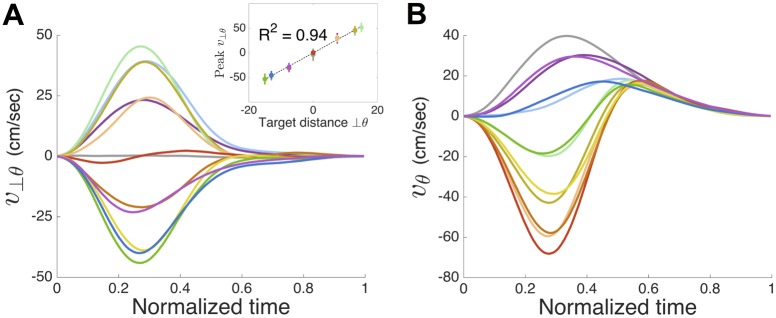
Timing of the velocity peak in Experiment 1. **(A)** Velocity component perpendicular to the visibility modulation direction (*θ*) against normalized time for the 12 directions of movement (colors as in Fig 4A). Inset shows peak speed against distance to target perpendicular to *θ*. **(B)** as (A) for the velocity component in the visibility of modulation direction.

We confirm these predictions by showing that timing of the velocity peak orthogonal to *θ* (*v*_⊥*θ*_) occurs at a similar time (Fig 6A; F_9,63_ = 0.840; p = 0.582 ANOVA on targets with non-zero distance) and the peak scales with the distance to the target in that direction (Fig 6A inset; R^2^ = 0.94). In contrast, the peaks occur at different times (Fig 6B; F_9,63_ = 11.022.; p<0.001) for velocity component along *θ* (*v*_*θ*_). This shows that indeed the qualitative features of the model are supported and that a feedforward component has an important role.

### Experiment 2: Variations in visibility sensitivity

To examine the sensitivity of the control process to changes in the visibility sensitivity, rather than direction of movement relative to the visibility modulation direction, we performed a second experiment in which we varied the visibility sensitivity across three levels.

**Fig 7:**
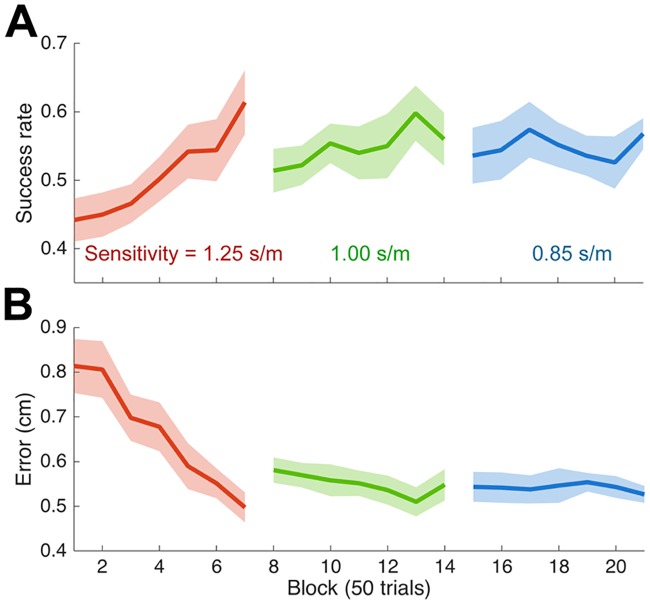
Performance in Experiment 2. **(A)** Success rate across blocks (mean ± s.e. across subjects) for the three visual sensitivities. **(B)** Error in the same format as (A).

Subjects made right to left transverse movements in the horizontal plane to a single target with three different visibility sensitivities. The visibility modulation direction was set to 270°, that is the direction towards the body. In three sequential blocks of 350 movements the visibility sensitivity was decreased (making the task increasingly difficult). As shown in Fig 7, success rate rose steadily and the error decreased with experience of the visibility field. There was a decrease in successes rate and increase in error when the visibility sensitivity was first decreased. On the second decrease in visibility sensitivity there was only a minimal change in performance.

**Fig 8:**
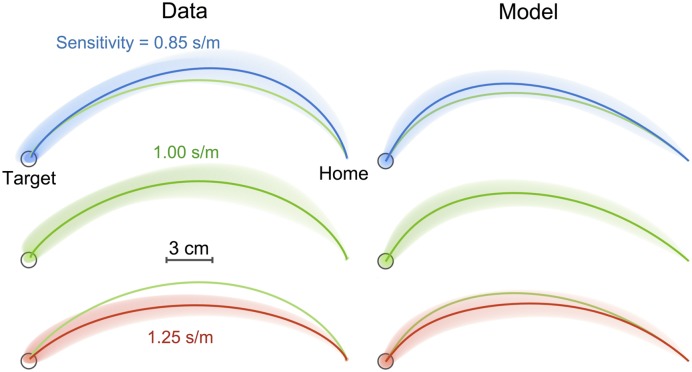
Hand paths in Experiment 2. Hand paths averaged over the final 50 movements of each sensitivity phase (subject mean ± s.d.). Left and right columns show the observed and simulated paths. For comparison purposes, the average paths for the intermediate sensitivity (green) is repeated on the high and low sensitivity path.

Examining the last 50 movements of each sensitivity session showed that the movements were curved, and deviated away from the body to move towards the body towards the end of the movement thereby increasing late visibility (Fig 8: left column). Importantly the curvature of the movement depended on the sensitivity, with an increasing curvature for lower sensitivity (the average path for the intermediate sensitivity is repeated on the high and low sensitivity plots). A repeated measures ANOVA on the maximum deviation of the path from a straight line showed a significant main effect of sensitivity (F_2,18_ = 42.9; p<0.001), with post hoc tests (Turkey’s HSD) demonstrates that the lowest sensitivity has the greatest deviation (p<0.001) and the highest sensitivity the smallest (p<0.001). This suggests that, when the sensitivity was lower, the movement was controlled to have a higher velocity in the direction of the visibility modulation direction.

**Fig 9:**
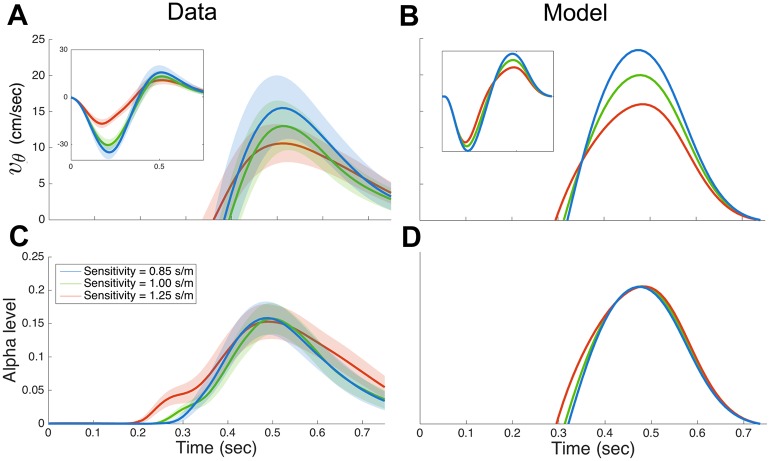
Temporal profiles of velocity in the visibility modulation direction and the corresponding alpha level in Experiment 2. **(A)** Velocity in the visibility modulation direction for the three visibility sensitivities. Data shown mean ± s.d. across the last 50 trials of each sensitivity session. Inset shows the entire movement and the main figure is clipped to the positive velocities when the cursor was visible. **(C)** Alpha level for the three visibility sensitivities. Data shown mean ± s.d. across the last 50 trials of each sensitivity session. (**C**)-(**D**) same as (**A**)-(**B**) for the model (mean only).

We examined the temporal profile of both the velocity in the visibility modulation direction (*v*_*θ*_) and the corresponding alpha level (Fig 9). Although the velocity profiles show variation with sensitivity (Fig 9A inset) the alpha level appear more similar during the late part of the movement (Fig 9B). These observations suggest that subjects may have made movements with different curvatures in order to regulate the visibility profile to be invariant across different visibility levels. Since *v*_*θ*_ and the alpha level show stereotypical bell-shaped profiles in the latter part of the movement, the statistics of their shapes can be summarized by their peak values. We performed repeated-measures ANOVAs on the peak *v*_*θ*_, peak alpha level, and the time of the peak with factor of sensitivity (3 levels). There was a significant effect of sensitivity on the peak velocity (F_2,8_ = 32.4, p<0.001), but no significant effect on either the peak alpha level (F_2,8_ = 0.596, p = 0.574) or the time of the peak (F_2,8_ = 1.557, p = 0.268). Pairwise planned comparisons on peak velocity with Bonferroni’s correction showed significant effects between high and medium (p = 0.003) and between high and low (p<0.001) sensitivity.

We examined predictions of the optimal control model under the three different visibility sensitivities. The trajectories (Fig 8, right column) are similar to the observed data, producing a higher curvature in lower sensitivity with a similar level of variability. In addition, simulated temporal profiles of velocity and the alpha level (Fig 9C and 9D) are also similar to the data, predicting a tighter overlap in the alpha level than velocity. This suggests that the optimal control model also exhibits the observed tendency to regulate the alpha level profile in the later phase of the movement.

### Sensitivity analysis

To examine the robustness of the optimal control model’s predictions to the choice of model parameters, we conducted a sensitivity analysis on the 24 parameters (8 main parameters that were used in the simulation and 16 potential parameters that were assumed to be zero or to be same as one of the 8 main parameters) used in the model. We sampled 10^5^ parameter sets from a uniform distribution within a hypercube in the 24 dimensional parameter space that spans ± 50% of the original values and for each sampled parameter set, we calculated the predicted trajectories for 12 movements of Experiment 1 (see “Sensitivity analysis” in Materials and Methods for details).

**Fig 10:**
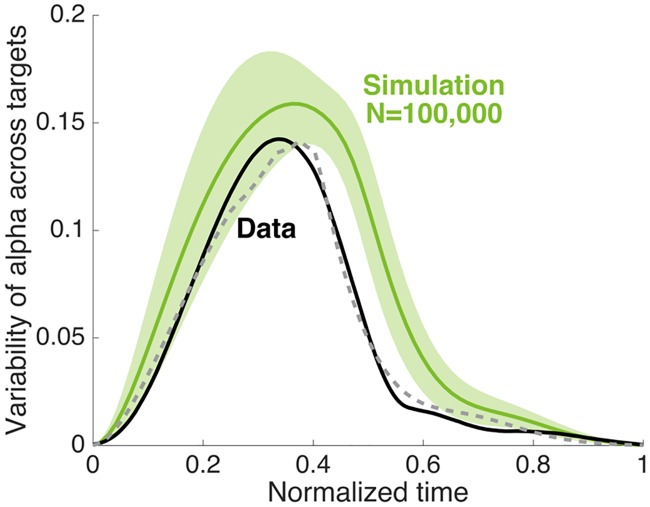
Parametric sensitivity of the model. Temporal profile of the variability of the alpha level in Experiment 1. Data (black) is mean across subjects (same as Fig 4D). For the simulation (green) we performed 10^5^ simulations with different parameter settings in the model and for each simulation we calculated the variability across the 12 directions for each time point and plot the mean ± s.d. of these variabilities. The grey dashed line is the single simulation which is closest to the data (MSE).

First, we tested if the model has a consistent tendency of regulating the late visibility of the movement. To test this, we analyzed how the variability of alpha level across directions changes over time. For each sampled parameter set, we simulated the expected mean profile of alpha level per movement direction and calculated the time profile of the standard deviation across all twelve directions. This average variability and standard deviation across all 10^5^ parameter sets were then compared with the one obtained from the data (variability across mean alpha level of all subjects, shown in Fig 4D). The result in Fig 10 (green) shows that the variability is substantially reduced after ~70% of the movement, which indicates that our optimal control model has an intrinsic property of regulating the late visibility of the movement. This overall pattern is also comparable with the variability profile computed from the data (black). The dashed grey line in Fig 10 is the single simulation from the 10^5^ which best matches the data showing that there are parameters which can account for the empirical data. Note that we have not fit the model to our data but simply show a good qualitative match using standard parameters.

## Discussion

We have examined how state-dependent sensory uncertainty affects the optimal planning of movements. Our results show that the inclusion of such uncertainty requires both a feedback controller, as is typical, but also the addition of a feedforward controller. We performed two experiments to examine whether movement planning is sensitive to state-dependent sensory variability during movement. Subjects were presented with a velocity-dependent visibility field in which the visibility of the hand depended on the speed of movement in a given direction. Subjects learned to make curved movement that improved the accuracy of the movements, and prioritized visibility in the latter portion of the movements near the target. The second experiment demonstrated that the planned movements were sensitive to subtle changes in the sensory feedback so that subjects produced movements with higher curvature in order to obtain similar levels of visual feedback. The results of both experiments were well captured by our optimal control model with state-dependent sensory noise. Together this work demonstrates that motion planning takes into account the sensory feedback that it will receive as a consequence of the action and our model can integrate active sensing for planning within a single optimal control framework.

We can analyze the structure of the optimal combined feedback and feedforward controllers to get some insight into their roles. We show (see S1 Text) that, when the system dynamics decouple into sub-systems (e.g. linear dynamics in two dimension), the resultant optimal controller can also be decoupled into controllers working in each sub-system. Based on the result, we also show that, while the feedback controller contributes to all sub-systems, working as a typical optimal feedback controller, the feedforward controller only controls the sub-system in which sensory feedback is affected by state-dependency (i.e. visibility modulation direction). This suggests that the role of the feedforward component is to generate a pre-planned action to gather sensory information, which is then exploited by the feedback controller to generate goal-directed movement. We also provided evidence that suggests that this decoupling strategy is seen in our empirical data.

Although our formalism falls within the active sensing framework it corresponds to a motor-centric view of active sensing. In our task, the goal is formulated in terms of accuracy and energy costs and the active sensing, in the form of altering the route to the goal, is only performed to obtain a better state estimate relevant for the motor task. Many active sensing paradigms have instead focused on active sensing where the only role for the motor system is to extract information for a purely sensory or cognitive task [18–21]. In these tasks the quality of some perceptual or cognitive inference is the primary goal and action subserves this goal by efficiently sampling the sensory world [9]. In contrast, in motor tasks the goal is to minimize motor-related costs and active sensing arises as these costs will also depend on how one senses the world and therefore how we move. As such, we show that the optimal control framework can be solved under state-dependent sensory variability and that using an iterative algorithm we can find the optimal controller. This controller trades off the benefits of increasing motor costs at some points in a movement to improve the state estimate which benefits later parts of a movement. As such the framework we have developed does not require a separate solution for estimation and control, but instead jointly solves them to produce the optimal combined feedback and feedforward controller.

The formalism we have developed has allowed us to examine affine state-dependent sensory variability. We have shown that humans can learn to move near optimally under such artificially applied visual noise. Although we did not alter proprioception, we ensured that the task could not be solved without using vision by including a small random translation offset between hand and cursor on each trial. The normal sensory variability in proprioception and visual location of the limb is unlikely to be linear given the nonlinear properties of the arm, and have only been partially mapped [10–13]. We chose to reduce noise with movement speed in particular directions and indeed studies have shown that without movement the perceived location of the limb can drift and that action can reset the perceived position [22]. For mathematical tractability, we approximated the system dynamics as linear, and it is important to note that, such a clean separation of the feedback and feedforward controllers is unlikely to be seen given a non-linear system such as body dynamics. We needed to make the linear approximation as the problem of finding an optimal control policy for a general nonlinear and non-Gaussian system, which is typically framed as a “partially observable Markov decision process” in the reinforcement learning literature, is often intractable [23]. To even get a near-optimal solution requires such problems to be simplified [for a review see 24]. Therefore, we have shown that people can be sensitive to state-dependent variability in sensory input and the extent to which the sensorimotor system takes the fine details of natural state-dependency into account is an open question. Our framework provides a starting point to address such questions.

Our focus has been on the reliability of sensory feedback and in particular how it might change with the state of the limb. This complements studies on the optimality of movement which have focused on altering sensory feedback by changing the relation between hand location and visual feedback so that straight movement became visually curved [25,26]. These show that participants tend to want to make visually straight line movements in the presence of such perturbations.

Our subjects made curved movements that improved their accuracy. By changing the curvature of the movement, the subjects could have improved visual feedback at any point throughout the movement. However, all subjects made movements that provided improved visual feedback towards the end of the movement, prior to reaching the target. This finding agrees with both modelling [27] and experimental results [28] showing that visuomotor feedback gains during movement peak around seventy percent of the movement distance. This also agrees with several lines of study that have shown that it is only towards the end of the movement that removal of visual feedback has a significant effect on the accuracy of the movements [29–31]. Although one might imagine that visual feedback at the very end of the movement could increase the accuracy further, both the delay for visual-based corrective responses (~150 ms [32]) and the extra cost of accelerating the arm for a corrective response [27] limit the efficacy and increase the cost of these later corrections. Within the framework of our model, improved visual feedback towards the end of the movement is optimal as the controller can rely on state estimates early in the movement, whereas noise gradually increases the state uncertainty as the movement progresses. Therefore, the optimal movement is one which provides the most accurate sensory feedback close to the end of the movement but with sufficient time for the corrective response to have an action.

Our results suggest that the sensory aspect of the movement has generally been overlooked within models that propose motor planning within the optimal control framework. A key novelty of our study is the evaluation of bidirectional influences between movement and sensation in a combined framework that links active sensing and optimal control. In addition, we provide experimental evidence that humans are able to learn an optimal sensorimotor strategy in the presence of state-dependent sensory variability and that optimal feedback control is insufficient in such a scenario as it requires a feedforward control component.

## Materials and Methods

### Ethics statement

The Cambridge Psychology Ethics Committee approved the study, and subjects gave written informed consent before participating.

### Experiment 1

Eight naive, right-handed subjects (aged 27.4 ± 4.2, 3 males) participated in the experiment. All subjects had normal or corrected-to-normal vision and had no reported neurological disorders. Subjects were seated in a chair with their shoulders restrained by a harness. The subject grasped the handle of a planar robotic manipulandum (vBOT) with their right hand [14]. Visual feedback was presented in the plane of the movement using a horizontally mounted monitor viewed through a mirror. The participant’s right forearm was supported by an airsled. The handle position was sampled at 1 kHz using optical encoders and visual feedback was updated at 60 Hz.

Subjects performed 15 cm center-in reaching movements towards a target (0.9 cm radius white circle) at the center of the workspace from twelve peripheral start positions (0.9 cm radius circles equally spaced at 30° intervals; Fig 1B). We chose center-in movements so as to provide extensive experience of the visibility field in the region around the single target. Piloting showed that performing center-out movement was much harder and required many more trials to learn. To start a trial, the hand was required to remain within the chosen start position for 0.75 s at which point the target appeared and a tone indicated that subjects should initiate the movement. Subjects made a reaching movement with the aim of placing the cursor (0.3 cm radius filled red circle) inside the target (position tolerance 0.6 cm). The movement was considered successful when the cursor was both within the target for 20 ms and its speed during this period was below 0.2 cm/s. Subjects were required to start the movement within 1 s of the tone (otherwise the trial was repeated) and to finish the movement within 3 s. Subjects received text feedback on the screen that depended on their movement duration measured from when they left the start position until they were within 0.6 cm of the target. The message displayed for durations of 0.5–0.95 s was “great”, for 0.2–0.5 or 0.95–1.25 s was “good”, and for durations less than 0.2 or 1.25–3 s was “too fast” or “timed out”, respectively. If longer than 3 s they received “missed” feedback.

At the end of each trial the vBOT passively moved the hand to the next start position along a minimum jerk trajectory [33]. During this return movement the cursor was extinguished. We wished to encourage subjects to rely on vision rather than on proprioception when reaching for the central target and therefore we added a random offset between the hand position and cursor on each trial. The offset was sampled uniformly over a 5 cm disk and the hand was passively moved back to a location so that the cursor appeared at the center of the starting target (i.e. the hand was offset from the starting position).

Two types of trials were used. On standard trials, the position of the hand was shown as a red filled circle (cursor) against a black background. On exposure trials, we controlled the visibility of the cursor by modulating its alpha level between 0 (invisible) and 1 (pure red as in standard trials). Similar to velocity-dependent force fields that are widely used in motor learning studies [15], we used a velocity-dependent visibility field in which the alpha level of the cursor depended on the velocity of the hand (*v*_*x*_, *v*_*y*_). Specifically the alpha level was related to the speed of the hand (*v*_*θ*_) in a fixed direction *θ*, where *v*_*θ*_ = *v*_*x*_*cosθ* + *v*_*y*_*sinθ* and the visibility, alpha, was set as:

$$\text{alpha}=\;\{\begin{array}{ll} 0, & \kappa v_{\theta }<0\\ \kappa v_{\theta }, & 0\leq \kappa v_{\theta }\leq 1\\ 1, & \kappa v_{\theta }>1 \end{array},$$ (1)

Where κ is the visibility sensitivity parameter and *θ* is termed visibility modulation direction as changes in speed in this direction modulates the alpha level. An example of the variation in the alpha level for different velocities is shown in Fig 1A for *θ* = 0°. In this example, motion in the leftward direction leads the cursor being invisible whereas the cursor becomes increasingly visible as hand speed increased in the rightward direction. For each subject, the visibility modulation direction was set to a single value corresponding to either rightward (0°), forward (90°), leftward (180°) or backward (270°). The eight subjects were randomly assigned to the four directions so that each direction was experienced by two subjects. The sensitivity κ was set to 1 s/m so that the cursor is only fully visible when the component of hand speed in the visibility modulation direction exceeds 1 m/s. This means that for a typical movement the cursor would be dim (or invisible) during the movement but with a visibility that modulates with speed. The cursor visibility for straight line minimum jerk trajectories for *θ* = 0° is shown in Fig 1B (for clarity displayed on a white background). As the visibility of the cursor is controlled by the alpha level, we use the terms “alpha level” and “visibility” interchangeably. In addition, we use the symbol *θ* and *v*_*θ*_ to denote the visibility modulation direction and the speed in the visibility modulation direction respectively.

A block of trials consisted of one reach from each of the 12 starting locations in a pseudorandom order. Each subject performed 83 blocks (996 trials), consisting of 8 pre-exposure standard blocks and 75 blocks of exposure under a velocity-dependent visibility field. Subjects had a one-minute rest break every 16 blocks, and were also allowed to take additional breaks whenever they wished. The instruction provided to subjects prior to the experiment contained only minimal information required to perform the experiment, including a general description of the vBOT system and an explanation that the visibility of the cursor will depend on the hand movement during the session.

### Experiment 2

Ten new naive subjects (aged 27.2 ± 4.8, 3 males) participated in the second experiment, which was similar to the first but designed to examine the control process under different sensitivities of the visibility field as well as to control for potential effects of eye movements. We required subject to fixate the target throughout the reach and monitored eye movements at 1 kHz (Eyelink 1000 with a tower mount, SR Research). A trial was terminated if the gaze was more than 2 cm from the target. To assist subjects in placing their hand on the start position (which was in peripheral vision) a virtual spring (10 N/cm) was applied when the cursor was more than 0.5 cm away from the start position. This spring turned off and a tone indicated the start of the movement. The experiment was performed using the same vBOT system and the graphics environment, but the computer screen was mounted vertically in front of the subject to allow the incorporation of the eye tracker. The eye-tracker setup did not include an airsled so we immobilized the subject’s wrist with a splint fastened with Velcro straps to reduce the degrees of freedom.

Subjects made a single 21 cm transverse movement from right to left. The radius of the cursor and target were 0.5 cm and 1.25 cm respectively (tolerance was set to 0.75 cm). The direction of the visibility was set to 270° (i.e. downward on the screen corresponding to movements towards the body). The message displayed on the screen for durations of 0.5–1 s was “great”, for 0.4–0.5 or 1–1.9 s was “good”, and for durations less than 0.4 or greater than 1.9 s was “too fast” or “too slow”, respectively. As in Experiment 1, we applied a random uniform offset but within a 2 cm disk.

After a brief familiarization and calibration for the eye tracker, subjects performed three phases of the experiment with 350 trials in each phase (1050 trials in total). The visibility sensitivity varied between the phases (*κ*: high–1.25, middle–1 and low–0.85 s/m). Subjects started with the high sensitivity block and then proceeded to the middle and then the low sensitivity blocks because simulations suggested that solutions for high sensitivities would be highly sub-optimal for low sensitivities whereas strategies for low sensitivity would often work for high. This encourages adaptation, which is often slow for changes from high variance tasks to low variance tasks [e.g. 34].

### Data analysis

Data were analyzed using MATLAB R2015a and statistical tests were performed using SPSS 22. Statistical significance was considered at the p<0.05 level for all tests.

To examine trajectories, we sampled 300 points uniformly over time from the start to the end of the movement. We then calculated mean positions and standard deviation ellipses for each sample across a set of trajectories of interest and displayed the mean trajectory with the envelope of the ellipses. To summarize time profiles of the velocities and the alpha levels, we first calculated the per-subject mean and standard deviation at each time point, and averaged them respectively across the subjects. This results in a time profile of the mean with the average standard deviation.

Learning was evaluated by two measures: success rate and error. For each block of 12 trials, success rate was measured by the ratio between successful trials and total trials, and error was measured by the distance between the target and the final cursor position. Statistical tests were performed on each measure to detect changes. For Experiment 1, we defined three epochs, late pre-exposure (blocks 6–8), early exposure (blocks 9–11) and late exposure (blocks 81–83), performed repeated-measures ANOVAs to compare different epoch.

### Optimal control model

We formulated the task within the framework of optimal control. We modeled the task as a linear dynamical system with a linear observation model that are both affected by Gaussian noise. We regarded the visibility of the cursor as affecting the observation noise in a state-dependent manner, with lower visibility equated to increased noise. We used a standard quadratic cost function to evaluate the control performance. Therefore, the problem of finding a control policy that minimizes the expected cost falls within the category of LQG (Linear Quadratic Gaussian) control. Here we provide a brief sketch of the general LQG formulation and the solution. To reduce the degrees of freedom of the model we also made several simplifying assumptions. For the more general case and further details see S1 Text.

#### System dynamics

We model the motion of the hand/cursor as a discrete-time linear dynamical system with state transition matrix *A* and control influence matrix *B*. We include both additive signal-independent and signal-dependent noise as is commonly assumed in the biological motor control [35]. Taken together, the system dynamics are governed by:

$$x_{t+1}=\;A\;x_{t}+\;B\left(I+F_{t}\right)\;u_{t}\;+\xi_{t}$$ (2)

where *x*_*t*_ and *u*_*t*_ are the state and the control at time *t* respectively. The state vector *x*_*t*_ had a dimension of 10 that includes a the Cartesian position (2 dimensions) and velocity (2 dimensions) of the cursor in the two dimensional plane; Cartesian forces (2 dimensions) applied to the handle; Cartesian muscle intermediate states (2 dimensions) for second order muscle-like low-pass filter converting the muscle control signal *u*_*t*_ to the force applied to the handle [36]; and the desired final Cartesian position (2 dimensions) of the cursor, which is added for notational convenience [17].

The motor command *u*_*t*_ is two dimensional representing control inputs to the muscles that act in Cartesian coordinates. The dynamics of the system are affected by signal-dependent noise applied to each motor command (*F*_*t*_ is a 2 × 2 matrix of which each element is a zero-mean normal distribution). The signal-independent noise component *ξ*_*t*_ is a multivariate random variable that follows a normal distribution with zero means and a diagonal covariance *Ω*^*ξ*^, i.e., each element of the noise is a scaled version of a unit normal distribution.

#### Observer

The controller does not have direct access to the state *x*_*t*_ but only to the sensory input *y*_*t*_ which is a 6 dimensional vector with Cartesian position (2), velocity (2) and force (2) given by the linear output model:

$$y_{t}\;=\;H\;x_{t}+\;g_{t}\left(a+\kappa d^{T}x_{t}\right)\;+\omega_{t}.$$ (3)

Similar to the system dynamics, we include both state-dependent and state-independent noise in the observation. To incorporate state-dependent noise we set *g*_*t*_ to be a vector with elements that are each drawn from a zero-mean normal distribution, and *a* to be a constant scaling factor, and *d* to be a unit vector defining the state-dependency, which is scaled by the sensitivity *κ* used in our experiment. To model our velocity-dependent visibility (Eq 1), we assumed that decreasing the visibility of the cursor could be approximated in the model by increasing the noise on the sensory feedback. For analytic tractability we chose the noise to be Gaussian. Therefore, *d* were set so that noise is sensitive to the velocity in the visibility modulation direction, and uncertainty increases with decreasing alpha and in particular the standard deviation of the noise has a negative linear relation with alpha (i.e. *d*^*T*^*x*_*t*_ = −*v*_*θ*_). We do not equate alpha levels of 0 with infinite noise as proprioception is still available. In addition, we assumed that when velocity exceeds a threshold at which *a* + *κd*^*T*^*x*_*t*_ becomes zero, the cursor is fully detectable with no state-dependent noise. However, the above affine state-dependent noise predicts that at velocities in the visibility direction above this value noise would increase again in the model. In order to prevent this, we made the observer switch between the above affine form and a constant form (i.e. without affine term) around this threshold. Although this made the system nonlinear, we found that our iterative algorithm, which applies this switching for each iteration, still stably reduced the cost (see S1 Text). Again signal-independent noise *ω*_*t*_ is assumed to follow the normal distribution with a diagonal covariance *Ω*^*ω*^.

We found that the inclusion of the signal-independent noises, *ξ*_*t*_ and *ω*_*t*_, (which required setting 10 and 6 parameters) had little effect on the solution and therefore we set these noise terms either to be zero (*ξ*_*t*_) or to be very small (all diagonal variances of *ω*_*t*_ were set to 10^−12^) in our simulations, where small variances in the signal-independent observation noise do not affect the result but add extra damping to the system that prevents ill-conditioned matrix inversions. In addition, to reduce the parameters of the signal-dependent noise *F*_*t*_, we set each of the terms within each matrix to have equal variance thereby reducing each signal-dependent matrix to a single parameter. Similarly, the state-dependent noise was only added to the sensory output terms corresponding to the position of the hand (i.e. only elements in *g*_*t*_ that correspond to the position had non-zero variance). However, we examined the sensitivity of the solution to inclusion of non-zero (or substantially greater than zero) *ξ*_*t*_ and *ω*_*t*_, different variances in *F*_*t*_, and non-zero elements in *g*_*t*_ that correspond to the velocity sensation (see “Sensitivity analysis” below).

#### Optimal control model

We formulate a finite horizon stochastic optimal control problem for the above system. Starting from a distribution of the initial state ${\hat{x}}_{1}=N\left(x_{1},\Sigma_{1}\right)$, the controller sequentially generates *n-1* control signals [*u*_1_, *u*_2_, …, *u*_*n*−1_] until the system reaches the final state *x*_*n*_. We define a quadratic cost induced by the controller:

$$Cost\left(u\right)=x_{n}Q_{n}x_{n}+\Sigma_{t=1}^{n-1}(x_{t}^{T}Qx_{t}+\;u_{t}^{T}Ru_{t}),$$ (4)

where the first term enforces the task goal by penalizing the final error and the term within the sum enforces regularization of the state and the control (e.g. smoothness or energy efficiency). As usually assumed in previous studies [4,27,37], we assumed *Q* to be zero and *R* to be diagonal. The optimal controller *u* = [*u*_1_, …, *u*_*n*−1_] is a control sequence that minimizes the cost:

$$\begin{array}{l} u=argmin\;E[Cost\left(u\right)|\;x_{1},\;\Sigma_{1}]. \end{array}$$ (5)

Since the system has uncertainty, the optimal controller contains a feedback term, which means that control for each step is determined based on an estimate of state. As the state is not directly observable, optimal control is accompanied by an optimal state estimator. In LQG, the optimal state estimator updates the estimated state $\hat{x}$ from the noisy observation *y* and is represented with the following linear dynamical system:

$${\hat{x}}_{t+1}=A\;{\hat{x}}_{t}\;+\;B\;u_{t}\;+\;K_{t}\;\left(\;y_{t}\;-\;H\;{\hat{x}}_{t}\right)\;+\eta_{t},$$ (6)

where first two terms are known dynamics of the system, followed by a corrective term based on the discrepancy between the actual (*y*_*t*_) and the expected ($H\;{\hat{x}}_{t}$) observation and an internal estimation noise (*η*_*t*_). The gain *K*_*t*_ is called the Kalman filter gain. As we assume that all the parameters from Eqs (2), (3), (4) and (6) are known a priori except *u*_*t*_ and *K*_*t*_, the optimal control and estimation problem requires computing an optimal controller *u*_*t*_ and a Kalman filter gain *K*_*t*_ for each step.

Our algorithm is based on the work of Todorov [17]. In accordance with the classical solution to the LQG with signal-independent noise, the algorithm consists of two procedures that recursively updates *u*_*t*_ and *K*_*t*_. While the classical solution regards these two procedures to be independent—and this is actually guaranteed by the separation theorem [38] for additive noises—this is no longer true for signal- and state-dependent noise. Therefore, an algorithm is used that alternately updates *u* and *K* until convergence. The algorithm is guaranteed to reduce the total cost for every iteration and therefore is expected to converge to the optimal solution. The original derivation [17] deals with both signal-dependent noise and state-dependent observation noise, but the affine term that resides in our state-dependent observation noise results in an optimal controller of the following affine form:

$$u_{t}=\;-L_{t}\;{\hat{x}}_{t}-l_{t}.$$ (7)

Importantly, the resultant controller contains both feedback ($L_{t}\;{\hat{x}}_{t}$) and feed-forward (*l*_*t*_) terms, while traditional LQG assumes a purely feedback controller (i.e. *l*_*t*_ = 0). Full details of the derivation with the resultant update rules for the controller (*L*_*t*_ and *l*_*t*_) and the Kalman gains and the model of two dimensional arm movement and corresponding parameters used in our simulation are provided in S1 Text.

### Sensitivity analysis

To examine the robustness of the optimal control model’s predictions to the choice of model parameters, we examined its sensitivity to parameter variation. The model has eight parameters: two for the state-dependent sensory noise (*a* and *g*_*t*_ in Eq 3), three weighting parameters for the cost function *Q*_*n*_ and *R*, two for the signal-dependent motor noise *F*_*t*_ and the internal noise *η*_*t*_, and one for the time constant of the muscle dynamics (see S1 Text). In addition, we added 16 additional parameters that for simplicity we had either set to zero or had set to be equal within a matrix (zero elements in *ξ*_*t*_, *ω*_*t*_ and *η*_*t*_; parameters for signal- and state-independent noise and internal noise, and zero elements and uniform variances in *F*_*t*_ and *g*_*t*_; variances for signal- and state-dependent noise). In this 24 dimensional parameter space, we defined a hypercube spanning each dimension and sampled 10^5^ parameter sets from a uniform distribution inside the hypercube. The range of the hypercube in each dimension was set to ±50% of the original values for non-zero parameters and fixed ranges for the parameters set to zero (3 cm, 3 cm/s and 0.03 N for position, velocity, and force related noise). For each sampled parameter set, we calculated the predicted trajectories for the 12 movements of Experiment 1.

## Supporting Information

S1 Text This supplementary material includes: 1) Full details of the derivation with the resultant update rules for the optimal controller and the Kalman gains. 2) Specific properties of the optimal controller for a system with decoupled dynamics. 3) The model of two dimensional arm movement and corresponding parameters used in our simulation. 4) Sensitivity and convergence analysis.
(PDF)
