# Correction: When Optimal Feedback Control Is Not Enough: Feedforward Strategies Are Required for Optimal Control with Active Sensing

**DOI:** 10.1371/journal.pcbi.1005370

**Published:** 2017-02-03

**Authors:** The PLOS Computational Biology Staff

There is an error in the legend for Figure 9. "(C)-(D) same as (A)-(B) for the model (mean only)" should instead read "(B),(D) same as (A),(C) for the model (mean only)".

The publisher apologizes for this error.
