# Supplementary material for: When Optimal Feedback Control Is Not Enough: Feedforward Strategies Are Required for Optimal Control with Active Sensing
Source: PLoS Comput Biol. 2016 Dec 14;12(12):e1005190. doi: 10.1371/journal.pcbi.1005190 (PMC5156370; doi:10.1371/journal.pcbi.1005190)
Supplement: S1 Text — This supplementary material includes: 1) Full details of the derivation with the resultant update rules for the optimal controller and the Kalman gains. 2) Specific properties of the optimal controller for a system with decoupled dynamics. 3) The model of two dimensional arm movement and corresponding parameters used in our simulation. 4) Sensitivity and convergence analysis. (PDF) [file pcbi.1005190.s001.pdf]

S1 Text: Supplementary Materials for

When optimal feedback control is not enough: feedforward strategies are required for optimal control with active sensing

Sang-Hoon Yeo, David W. Franklin and Daniel M. Wolpert

1 Optimal Controller

We start from the control-observation model with signal-dependent and state-dependent noise (see Main text for details of this formulation):

$$\begin{aligned}
&\textit{Dynamics} && x_{t+1} = Ax_t + B(I + F_t)u_t + \xi_t \\
&\textit{Observer} && y_t = Hx_t + g_t(a + \kappa d^\top x_t) + \omega_t \\
&\textit{Cost per step} && x_t^\top Q x_t + u_t^\top R u_t \\
&\textit{Final cost} && x_n^\top Q_n x_n.
\end{aligned}$$

The state of the system x is estimated as $\hat{x}$ by a Kalman filter.

$$\hat{x}_{t+1} \quad = \quad A\hat{x}_t + Bu_t + K_t(y_t - H\hat{x}_t) + \eta_t,$$

where the initial mean and covariance of the estimated state, $\hat{x}_1$ and Σ_1 are given. Now, we assume that the cost-to-go function $v_t(x_t, \hat{x}_t)$ can be represented by the following "affine-quadratic" form:

$$v_t(x_t, \hat{x}_t) = x_t^\top S_t^x x_t + e_t^\top S_t^e e_t + s_t^{x\top} x_t + s_t, \tag{1}$$

where $e = x - \hat{x}$ and the final conditions are given as $S_n^x = Q_n,\ S_n^e = 0,\ s_n^x = s_n = 0$. Here we use the term "affine-quadratic" to distinguish it from the conventional quadratic form that has no linear term $s_t^{x\top} x_t$.

Now, assume that the system is optimally controlled for $t = [t+1, \cdots, n]$ and an optimal feedback policy $u_t = u_t(\hat{x}_t)$ is given. Then the corresponding cost-to-go function $v_t(x_t, \hat{x}_t)$ should satisfy the following Bellman equation:

$$v_t(x_t, \hat{x}_t) = x_t^\top Q x_t + u_t^\top R u_t + \mathrm{E}[v_{t+1}(x_{t+1}, \hat{x}_{t+1}) | x_t, \hat{x}_t, u_t]. \tag{2}$$

To represent the last expectation term with variables at time t, we use the following dynamics

of x, $\hat{x}$ and e:

$$\begin{aligned}
x_{t+1} &= Ax_t + B(I + F_t)u_t + \xi_t \\
e_{t+1} &= x_{t+1} - \hat{x}_{t+1} \\
&= (A - K_t H)e_t - K_t g_t(a + \kappa d^\top x_t) + BF_t u_t + \xi_t - \eta_t - K_t \omega_t,
\end{aligned}$$

and their conditional means and covariances:

$$\begin{aligned}
\mathrm{E}[x_{t+1}|x_t, \hat{x}_t, u_t] &= Ax_t + Bu_t && (3) \\
\mathrm{E}[e_{t+1}|x_t, \hat{x}_t, u_t] &= (A - K_t H)e_t && (4) \\
\mathrm{Cov}[x_{t+1}|x_t, \hat{x}_t, u_t] &= \mathrm{E}[BF_t u_t u_t^\top F_t^\top B^\top |u_t] + \Omega^\xi \\
\mathrm{Cov}[e_{t+1}|x_t, \hat{x}_t, u_t] &= \mathrm{E}[BF_t u_t u_t^\top F_t^\top B^\top |u_t] + \Omega^\xi + \Omega^\eta + K_t \Omega^\omega K_t^\top \\
&\quad + K_t \ \mathrm{E}[g_t(a + \kappa d^\top x_t)(a + \kappa d^\top x_t)^\top g_t^\top |x_t] \ K_t^\top.
\end{aligned}$$

Given that BF_t consists of mutually independent random variables $[\varepsilon_t^1, \cdots, \varepsilon_t^c]$ each of which follows a unit normal distribution (i.e. $\varepsilon_t^i \sim \mathcal{N}(0, 1)$), $BF_t u_t$ can be decomposed into $BF_t u_t = \varepsilon_t^1 C_1 u_t + \cdots + \varepsilon_t^c C_c u_t$, where C_i's are matrix bases. Similarly, assuming that G_t consists of d normally distributed random varilables $[\epsilon_t^1, \cdots, \epsilon_t^d]$, the same decomposition can be applied to $g_t(a+\kappa d^\top x_t)$, which results $g_t(a + \kappa d^\top x_t) = \epsilon_t^1(a_1 + D_1 x_t) + \cdots + \epsilon_t^d(a_d + D_d x_t)$. Note that κ is now included in D. Using these, the covariances become

$$\begin{aligned}
\mathrm{Cov}[x_{t+1}|x_t, \hat{x}_t, u_t] &= \sum_{i=1}^{c} C_i u_t u_t^\top C_i^\top + \Omega^\xi && (5) \\
\mathrm{Cov}[e_{t+1}|x_t, \hat{x}_t, u_t] &= \sum_{i=1}^{c} C_i u_t u_t^\top C_i^\top + \sum_{i=1}^{d} K_t(a_i + D_i x_t)(a_i + D_i x_t)^\top K_t^\top && (6) \\
&\quad + \Omega^\xi + \Omega^\eta + K_t \Omega^\omega K_t^\top.
\end{aligned}$$

Since the expected cost-to-go at time $t + 1$ is:

$$\mathrm{E}[v_{t+1}|x_t, \hat{x}_t, u_t] = \mathrm{E}[x_{t+1}^\top S_{t+1}^x x_{t+1} + e_{t+1}^\top S_{t+1}^e e_{t+1} + {s_{t+1}^x}^\top x_{t+1} + s_{t+1}],$$

where each element can be represented by variables at time t using Equation (3, 4, 5, 6)

$$\begin{aligned}
\mathrm{E}[x_{t+1}^\top S_{t+1}^x x_{t+1}|x_t,\hat{x}_t,u_t] &= x_t^\top A^\top S_{t+1}^x A x_t + u_t^\top B^\top S_{t+1}^x B u_t \\
&\quad +2x_t^\top A^\top S_{t+1}^x B u_t + \sum_{i=1}^{c} u_t^\top C_i^\top S_{t+1}^x C_i u_t + tr(S_{t+1}^x \Omega^\xi) \\
\mathrm{E}[e_{t+1}^\top S_{t+1}^e e_{t+1}|x_t,\hat{x}_t,u_t] &= e_t^\top (A-K_tH)^\top S_{t+1}^e (A-K_tH) e_t \\
&\quad + \sum_{i=1}^{c} u_t^\top C_i^\top S_{t+1}^e C_i u_t \\
&\quad + \sum_{i=1}^{d} (a_i + D_i x_t)^\top K_t^\top S_{t+1}^e K_t (a_i + D_i x_t) \\
\mathrm{E}[{s_{t+1}^x}^\top x_{t+1}|x_t,\hat{x}_t,u_t] &= {s_{t+1}^x}^\top (A x_t + B u_t) \\
\mathrm{E}[s_{t+1}|x_t,\hat{x}_t,u_t] &= s_{t+1}.
\end{aligned}$$

Putting these together, the cost-to-go function at time t can be written as

$$v_t = x_t^\top W_x x_t + u_t^\top W_u u_t + e_t^\top W_e e_t + 2x_t^\top W_{xu} u_t + {w_x}^\top x_t + {w_u}^\top u_t + w_0, \tag{7}$$

where

$$\begin{aligned}
W_x &= A^\top S_{t+1}^x A + \sum_{i=1}^{d} D_i^\top K_t^\top S_{t+1}^e K_t D_i + Q \\
W_u &= B^\top S_{t+1}^x B + \sum_{i=1}^{c} C_i^\top (S_{t+1}^x + S_{t+1}^e) C_i + R \\
W_e &= (A-K_tH)^\top S_{t+1}^e (A-K_tH) \\
W_{xu} &= A^\top S_{t+1}^x B \\
{w_x}^\top &= 2\sum_{i=1}^{d} a_i^\top K_t^\top S_{t+1}^e K_t D_i + {s_{t+1}^x}^\top A \\
{w_u}^\top &= {s_{t+1}^x}^\top B \\
w_0 &= \sum_{i=1}^{d} a_i^\top K_t^\top S_{t+1}^e K_t a_i + s_{t+1} \\
&\quad + tr\left(S_{t+1}^x \Omega^\xi + S_{t+1}^e \left(\Omega^\xi + \Omega^\eta + K_t \Omega^\omega K_t^\top\right)\right).
\end{aligned}$$

Then, the optimal policy is $u_t = -W_u^{-1}\left(W_{xu}^\top x_t + \frac{1}{2} w_u\right) = -L_t x_t - l_t$. Since the policy should depend on the observed state $\hat{x}$, not x, we take a conditional mean of the policy using $\mathrm{E}[x_t|\hat{x}_t] = \hat{x}_t$, which results $u_t = -L_t\hat{x}_t - l_t = -L_t(x_t - e_t) - l_t$. Applying this to the equation (7), we finally get

$$v_t = x_t^\top (W_x - W_{xu}L_t) x_t + e_t^\top (W_e + W_{xu}L_t) e_t + (w_x^\top - w_u^\top L_t) x_t - \frac{1}{2} w_u^\top l_t + w_0. \tag{8}$$

Together with $v_n = x_n^\top Q_n x_n$ and v_t, this is also in an affine-quadratic form and therefore completes the proof by induction that the cost-to-go function is always affine-quadratic. The corresponding update rule is

$$
\begin{aligned}
L_t &= \left(B^\top S^x_{t+1} B + \sum_{i=1}^{c} C_i^\top (S^x_{t+1} + S^e_{t+1}) C_i + R\right)^{-1} B^\top S^x_{t+1} A && (9)\\
l_t &= \frac{1}{2}\left(B^\top S^x_{t+1} B + \sum_{i=1}^{c} C_i^\top (S^x_{t+1} + S^e_{t+1}) C_i + R\right)^{-1} B^\top s^x_{t+1} && (10)\\
S^x_t &= A^\top S^x_{t+1} A + \sum_{i=1}^{d} D_i^\top K_t^\top S^e_{t+1} K_t D_i + Q - A^\top S^x_{t+1} B L_t && (11)\\
S^e_t &= (A - K_t H)^\top S^e_{t+1} (A - K_t H) + A^\top S^x_{t+1} B L_t && (12)\\
s^x_t &= \left(\sum_{i=1}^{d} 2 a_i^\top K_t^\top S^e_{t+1} K_t D_i + {s^x_{t+1}}^\top A - {s^x_{t+1}}^\top B L_t\right)^\top && (13)\\
s_t &= s_{t+1} + \sum_{i=1}^{d} a_i^\top K_t^\top S^e_{t+1} K_t a_i - \frac{1}{2} {s^x_{t+1}}^\top B l_t \\
&\quad + tr\left(S^x_{t+1} \Omega^\xi + S^e_{t+1}\left(\Omega^\xi + \Omega^\eta + K_t \Omega^\omega K_t^\top\right)\right), && (14)
\end{aligned}
$$

where the final conditions are $S^x_n = Q, S^e_n = 0, s^x_n = 0, s_n = 0$. Assuming $\hat{x}_1$ and $\mathrm{E}[e_1 e_1^\top] = \Sigma_1$ are known and $\mathrm{E}[e_1] = 0$, the total expected cost is

$$
\begin{aligned}
\mathrm{E}[v_1] &= \mathrm{E}[x_1^\top S^x_1 x_1 + e_1^\top S^e_1 e_1 + {s^x_1}^\top x_1 + s_1] \\
&= \mathrm{E}[(\hat{x}_1 + e_1)^\top S^x_1 (\hat{x}_1 + e_1) + e_1^\top S^e_1 e_1 + {s^x_1}^\top (\hat{x}_1 + e_t) + s_1] && (15)\\
&= \hat{x}_1^\top S^x_1 \hat{x}_1 + tr((S^x_1 + S^e_1)\Sigma_1) + {s^x_1}^\top \hat{x}_1 + s_1.
\end{aligned}
$$

2 Optimal Estimator

Now we determine the Kalman filter K_t that minimizes the expected cost-to-go function at each time step. As the cost-to-go-function is also affine-quadratic with respect to K_t, $\partial \mathrm{E}[v_t]/\partial K_t = 0$ is a necessary and sufficient condition for the minimum. K-dependent terms in v_t are

$$
\begin{aligned}
q(K_t) &= \sum_{i=1}^{d} x_t^\top D_i^\top K_t^\top S^e_{t+1} K_t D_i x_t + e_t^\top (A - K_t H)^\top S^e_{t+1} (A - K_t H) e_t \\
&\quad + \sum_{i=1}^{d} 2 a_i^\top K_t^\top S^e_{t+1} K_t D_i x_t + \sum_{i=1}^{d} a_i^\top K_t^\top S^e_{t+1} K_t a_i + tr\left(S^e_{t+1} K_t \Omega^\omega K_t^\top\right).
\end{aligned}
$$

Using the following matrix lemmas:

$$
\begin{aligned}
x^\top y &= tr(xy^\top) \\
\frac{\partial tr(KUK^\top V)}{\partial K} &= VKU + V^\top KU^\top \\
\frac{\partial x^\top K y}{\partial K} &= xy^\top,
\end{aligned}
$$

K_t that minimizes $\mathrm{E}[q(K_t)]$ is

$$
K_t = A\Sigma_t^e H^\top \left(\sum_{i=1}^{d} \left(D_i \Sigma_t^x D_i^\top + a_i \bar{x}_t^\top D_i^\top + D_i \bar{x}_t a_i^\top + a_i a_i^\top \right) + H\Sigma_t^e H^\top + \Omega^\omega \right)^{-1}, \tag{16}
$$

where Σ's are uncentered and unconditional covariances (i.e. $\Sigma_t^x = \mathrm{E}[x_t x_t^\top]$ and $\Sigma_t^e = \mathrm{E}[e_t e_t^\top]$) and $\bar{x}_t$ is the unconditional mean of x_t. Note that $\bar{x}_t$ represents the nominal trajectory, a trajectory that would be produced by controller if there are no noises.

Given that the dynamics of $e, x, \hat{x}$ are

$$
\begin{aligned}
e_{t+1} &= (A - K_t H)e_t - \sum_{i=1}^{d} \epsilon_t^i K_t (a_i + D_i x_t) - \sum_{i=1}^{c} \varepsilon_t^i C_t (L_t \hat{x}_t + l_t) + \xi_t - \eta_t - K_t \omega_t \\
x_{t+1} &= Ax_t - \left(B + \sum_{i=1}^{c} \varepsilon_t^i C_t \right) (L_t \hat{x}_t + l_t) + \xi_t \\
\hat{x}_{t+1} &= x_{t+1} - e_{t+1} \\
&= (A - BL_t)\hat{x}_t - Bl_t + K_t \left(He_t + \sum_{i=1}^{d} \epsilon_t^i (a_i + D_i x_t) \right) + \eta_t + K_t \omega_t,
\end{aligned}
$$

update rules for the unconditional means of $e, x, \bar{x}$ are

$$
\bar{e}_{t+1} = (A - K_t H)\bar{e}_t \tag{17}
$$

$$
\bar{x}_{t+1} = (A - BL_t)\bar{x}_t + BL_t \bar{e}_t - Bl_t \tag{18}
$$

$$
\bar{\hat{x}}_{t+1} = \bar{x}_{t+1} - \bar{e}_{t+1}, \tag{19}
$$

with initial conditions $\bar{e}_1 = 0, \bar{x}_1 = \bar{\hat{x}}_1 = \hat{x}_1$. Update rules for covariance Σ_t^e and Σ_t^x are

$$
\begin{aligned}
\Sigma_{t+1}^e &= (A - K_t H)\Sigma_t^e (A - K_t H)^\top \\
&\quad + \sum_{i=1}^{c} C_i (L_t \Sigma_t^{\hat{x}} L_t^\top + L_t \bar{\hat{x}}_t l_t^\top + l_t \bar{\hat{x}}_t^\top L_t^\top + l_t l_t^\top) C_i^\top \\
&\quad + \Sigma_t^n + \Omega^\xi + \Omega^\eta + K_t \Omega^\omega K_t^\top
\end{aligned} \tag{20}
$$

$$
\begin{aligned}
\Sigma_{t+1}^{\hat{x}} &= (A - BL_t)\Sigma_t^{\hat{x}} (A - BL_t)^\top + K_t H \Sigma_t^e H^\top K_t^\top \\
&\quad + (A - BL_t)\Sigma_t^{\hat{x}e} H^\top K_t^\top + K_t H \Sigma_t^{e\hat{x}} (A - BL_t)^\top \\
&\quad - (A - BL_t)\bar{\hat{x}}_t l_t^\top B^\top - B l_t \bar{\hat{x}}_t^\top (A - BL_t)^\top \\
&\quad - B l_t \bar{e}_t^\top H^\top K_t^\top - K_t H \bar{e}_t l_t^\top B^\top \\
&\quad + \Sigma_t^n + B l_t l_t^\top B^\top + \Omega^\eta + K_t \Omega^\omega K_t^\top
\end{aligned} \tag{21}
$$

$$
\begin{aligned}
\Sigma_{t+1}^{\hat{x}e} &= (A - BL_t)\Sigma_t^{\hat{x}e} (A - K_t H)^\top - B l_t \bar{e}_t^\top (A - K_t H)^\top \\
&\quad + K_t H \Sigma_t^e (A - K_t H)^\top - \Sigma_t^n - \Omega^\eta - K_t \Omega^\omega K_t^\top
\end{aligned} \tag{22}
$$

$$
\Sigma_t^x = \Sigma_t^e + \Sigma_t^{\hat{x}} + \Sigma_t^{\hat{x}e} + \Sigma_t^{e\hat{x}}, \tag{23}
$$

where

$$
\Sigma_t^n = \sum_{i=1}^{d} K_t (a_i a_i^\top + a_i \bar{x}_t^\top D_i^\top + D_i \bar{x}_t a_i^\top + D_i \Sigma_t^x D_i^\top) K_t^\top .
$$

Initial conditions are $\Sigma_1^e = \Sigma_1, \Sigma_1^{\hat{x}} = \hat{x}\hat{x}^\top, \Sigma_1^{\hat{x}e} = 0$.

3 Switching between affine and constant form

One problem of the suggested affine state-dependency is that the noise will increase when $a_i + D_i x_t$ becomes negative. To prevent this, for each iteration, we simply set the affine term to be zero ($a_i = 0$ and $D_i = 0$) when $a_i + D_i \bar{x}_t$ becomes negative. We will show later (Figure S1) that the algorithm still stably converges to the solution.

4 Iterative Solver

As originally suggested by Todorov [1], the optimal solutions for both controller (L_t and l_t) and estimators (K_t) can be obtained by iterative updates that guarantee the convergence. Again, this guarantee does not hold any more in our case due to the switching behavior, but we will empirically show that the algorithm converges. The overall procedure is sketched in **Algorithm S1**.

Algorithm S1 Calculate $L_t,\ l_t,\ K_t$ for $t = [1, \cdots, n-1]$

procedure ITERATIVE SOLVER
  $L_t = 0, l_t = 0 \ \forall\ t = [1, \cdots, n-1]$ ▷ Controller initialization
  $K_t = 0 \ \forall\ t = [1, \cdots, n-1]$ ▷ Estimator initialization
  $E[v_1] \leftarrow Inf$
  do
    $(L_t, l_t) \leftarrow$ ESTIMATOR UPDATE (K_t)
    $K_t \leftarrow$ CONTROLLER UPDATE (L_t, l_t)
    Update $E[v_1]$ ▷ Equation(15)
    Switch state-dependent noise when necessary ▷ Section(3)
  while ($E[v_1]$ reduced)
end procedure

procedure CONTROLLER UPDATE(K_t)
  for $t = (n-1) : -1 : 1$ **do**
    Update $L_t \ \ l_t$ ▷ Equation(9, 10)
    Update $S_t^x \ \ S_t^e \ \ s_t^x \ \ s_t$ ▷ Equation(11, 12, 13, 14)
  end for
end procedure

procedure ESTIMATOR UPDATE(L_t, l_t)
  for $t = 1 : 1 : (n-1)$ **do**
    Update K_t ▷ Equation(16)
    Update $\bar{e}_{t+1} \ \ \bar{x}_{t+1} \ \ \bar{\hat{x}}_{t+1}$ ▷ Equation(17, 18, 19)
    Update $\Sigma^e_{t+1} \ \ \Sigma^{\hat{x}}_{t+1} \ \ \Sigma^{\hat{x}e}_{t+1} \ \ \Sigma^x_{t+1}$ ▷ Equation(20, 21, 22, 23)
  end for
end procedure

5 A system with de-coupled dynamics

To understand the characteristics of the suggested affine controller, we consider a typical case when the deterministic dynamics ($x_{t+1} = Ax_t + Bu_t$) of the system and the cost matrices (Q and R) are decoupled into k mutually independent sub-systems, i.e. when A, B, Q and R are all block-diagonal matrices with k-blocks of the same structure and therefore the whole system is represented as follows:

$$\begin{bmatrix} x^1_{t+1} \\ \vdots \\ x^k_{t+1} \end{bmatrix} = \begin{pmatrix} A^1 & & \\ & \ddots & \\ & & A^k \end{pmatrix} \begin{bmatrix} x^1_t \\ \vdots \\ x^k_t \end{bmatrix} + \begin{pmatrix} B^1 & & \\ & \ddots & \\ & & B^k \end{pmatrix} \begin{bmatrix} u^1_t \\ \vdots \\ u^k_t \end{bmatrix}, \quad Q = \begin{pmatrix} Q^1 & & \\ & \ddots & \\ & & Q^k \end{pmatrix}, \quad R = \begin{pmatrix} R^1 & & \\ & \ddots & \\ & & R^k \end{pmatrix}.$$

Let $\mathbb{S}_k$ be a group of all k-block-diagonal matrices (i.e. $A, B, Q, R \in \mathbb{S}_k$). Note that this group is closed under multiplication (i.e. if $X \in \mathbb{S}_k$ and $Y \in \mathbb{S}_k$, then $XY \in \mathbb{S}_k$). As will be shown in the

next section, our two-dimensional reaching experiment can be considered to be this case ($k = 2$). Now, let $\mathbb{U}_{\{j_1, j_2, \cdots\}}$ be a set of column vectors (in either state, control or observation space) that can only have non-zero elements in the entries that correspond to the blocks $j_1, j_2, \cdots$. In addition, let e_m be a standard basis vector (either in state, control or observation space) that has 1 at the m-th entry and 0 elsewhere, by which a set of all matrices that can only have a single non-zero element is represented as $\mathbb{B} = span(e_q e_r^\top) \;\; \forall\, q, r$. Based on these notations, we prove the following two theorems featuring important characteristics of the derived optimal controller.

First, it can easily be shown that:

Lemma 1. *if $P \in \mathbb{B}$, then $P^\top X P \in \mathbb{S}_k \;\forall$ valid X,*

and this leads to

Theorem 1. *If $A, B, Q, R \in \mathbb{S}_k$ and $C_i, D_i \in \mathbb{B} \;\forall i$, then $L_t \in \mathbb{S}_k \;\forall t = [1, \cdots, n-1]$.*

Proof. Remember the following update rule for L_t:

$$L_t = \left(B^\top S^x_{t+1} B + \sum_{i=1}^{c} C_i^\top (S^x_{t+1} + S^e_{t+1}) C_i + R \right)^{-1} B^\top S^x_{t+1} A. \qquad \text{(9 revisited)}$$

From the lemma above, $\sum_{i=1}^{c} C_i^\top (S^x_{t+1} + S^e_{t+1}) C_i \in \mathbb{S}_k$, and all other components except S^x_{t+1} belong to $\mathbb{S}_k$ by definition. If we assume that $S^x_{t+1} \in \mathbb{S}_k$, then $L_t \in \mathbb{S}_k$ and also $S^x_t \in \mathbb{S}_k$ by the update rule for S^x_{t+1}:

$$S^x_t = A^\top S^x_{t+1} A + \sum_{i=1}^{d} D_i^\top K_t^\top S^e_{t+1} K_t D_i + Q - A^\top S^x_{t+1} B L_t, \qquad \text{(11 revisited)}$$

where $\sum_{i=1}^{d} D_i^\top K_t^\top S^e_{t+1} K_t D_i \in \mathbb{S}_k$ by the lemma and all other components, including L_t, belong to $\mathbb{S}_k$. As $S^x_n = Q \in \mathbb{S}_k$, this completes the proof by induction. □

The above result shows that the resultant feedback controller $L_t x_t$ is always decoupled into k independent controllers, regardless of the couplings caused by signal-dependent or state-dependent noises.

Secondly, it can easily be shown that:

Lemma 2. *If $X \in \mathbb{S}_k$ and $y \in \mathbb{U}_{\{j1, j2, \cdots\}}$, then $Xy \in \mathbb{U}_{\{j1, j2, \cdots\}}$,*

and this results

Theorem 2. *If $D_i \in span(e_q e_r^\top)$ and $e_r \in \mathbb{U}_{\{j_i\}} \;\forall i = [1, \cdots, d]$, then $l_t \in \mathbb{U}_{\{j_1, \cdots, j_d\}} \;\;\forall t = [1, \cdots, n-1]$*

Proof. Consider the update rule for l_t:

$$l_t = \frac{1}{2} \left(B^\top S^x_{t+1} B + \sum_{i=1}^{c} C_i^\top (S^x_{t+1} + S^e_{t+1}) C_i + R \right)^{-1} B^\top s^x_{t+1}. \qquad \text{(10 revisited)}$$

From the above proposition, we have proved that the whole matrix on the lefthand side of s^x_{t+1} belongs to $\mathbb{S}_k$. Then using the lemma above, we can only show that $s^x_{t+1} \in \mathbb{U}_{\{j_1,\cdots,j_d\}}$. Remember the update rule for s^x_t is

$$s^x_t = \left(\sum_{i=1}^{d} 2a_i^\top K_t^\top S^e_{t+1} K_t D_i\right) + (A - BL_t)^\top s^x_{t+1}, \qquad \text{(13 revisited)}$$

and the final condition is $s^x_n = 0 \in \mathbb{U}_{\{j1,\cdots,jd\}}$. If $s^x_{t+1} \in \mathbb{U}_{\{j1,\cdots,jd\}}$, then $(A - BL_t)^\top s^x_{t+1} \in \mathbb{U}_{\{j1,\cdots,jd\}}$ from the lemma above, and for each i,

$$a_i^\top K_t^\top S^e_{t+1} K_t D_i = (a_i^\top K_t^\top S^e_{t+1} K_t e_q) e_r^\top \in \mathbb{U}_{\{ji\}}.$$

Therefore $s^x_t \in \mathbb{U}_{\{j1,\cdots,jd\}}$ too and this completes the proof by induction. □

This result shows that the feed-forward controller l_t only controls sub-system dynamics that affect the observation noise.

Taken together, if a given system dynamics is decoupled, the resultant optimal controller exhibits two important characteristics. First, the feedback part of the controller is decoupled, where each sub-controller independently drives the corresponding sub-state to the goal state. Second, on the other hand, the feed-forward part of the controller only generates control signals that affect (possibly reduces) the observation noise. Therefore, the feed-forward part can be considered as an offline, pre-planned motor program that collects the sensory information, and the feedback part can be considered as an online motor program that tries to achieve the given task goal based on the sensory information that is collected online.

6 Model of two dimensional reaching

The dynamics of the reaching movement was modelled as a linear two-dimensional dynamics. The model assumes a point-mass system to which the control signal is smoothened by a muscle-like second order low-pass filter. Deterministic dynamics and observer of the one-dimensional model are represented as follows:

$$\check{x}^i_t = \begin{bmatrix} p^i_t \\ v^i_t \\ f^i_t \\ g^i_t \\ p^{*i} \end{bmatrix}, \; \check{A} = \begin{pmatrix} 1 & \Delta & 0 & 0 & 0 \\ 0 & 1 & dt/m & 0 & 0 \\ 0 & 0 & 1-\Delta/\tau & \Delta/\tau & 0 \\ 0 & 0 & 0 & 1-\Delta/\tau & 0 \\ 0 & 0 & 0 & 0 & 1 \end{pmatrix}, \; \check{B} = \begin{pmatrix} 0 \\ 0 \\ 0 \\ \Delta/\tau \\ 0 \end{pmatrix}, \; \check{H} = \begin{pmatrix} 1 & 0 & 0 & 0 & 0 \\ 0 & 1 & 0 & 0 & 0 \\ 0 & 0 & 1 & 0 & 0 \end{pmatrix},$$

where p^i_t, v^i_t and f^i_t are the position, velocity and force of the hand in i^{th} dimension at time t, p^{*i} is the goal position, Δ is the time step (set to 0.01 s), m is the effective mass of the hand (set to 1

kg), and τ is time constant of the low-pass filter [1]. Corresponding cost matrices are given as

$$\check{R} = r,\ \check{Q} = O_{5\times5},\ \check{Q}_n = \begin{pmatrix} 1 & 0 & 0 & 0 & -1 \\ 0 & w_v^2 & 0 & 0 & 0 \\ 0 & 0 & w_f^2 & 0 & 0 \\ 0 & 0 & 0 & 0 & 0 \\ -1 & 0 & 0 & 0 & 1 \end{pmatrix},$$

where $O_{m\times n}$ represents a $m \times n$ zero matrix, r is a regularization factor, and w_v and w_f are relative weights of the penalties on non-zero final velocity and force, with respect to the positional penalty.

Now we build a two-dimensional system by stacking the above system:

$$x_t = \begin{bmatrix} \check{x}_t^1 \\ \check{x}_t^2 \end{bmatrix},\ A = \begin{bmatrix} \check{A} & O_{5\times5} \\ O_{5\times5} & \check{A} \end{bmatrix},\ B = \begin{bmatrix} \check{B}^1 & O_{5\times1} \\ O_{5\times1} & \check{B}^2 \end{bmatrix},\ H = \begin{bmatrix} \check{H}^1 & O_{3\times5} \\ O_{3\times5} & \check{H}^2 \end{bmatrix}.$$

Both systems use the same Δ and τ's. Similarly, cost matrices will be

$$R = \begin{bmatrix} r & 0 \\ 0 & r \end{bmatrix},\ Q = O_{10\times10},\ Q_n = \begin{bmatrix} \check{Q}_n & O_{5\times5} \\ O_{5\times5} & \check{Q}_n \end{bmatrix}.$$

Now we add noise elements to the system. For convenience, signal and state independent noise terms ξ_t, ω_t are both set to zero and the covariance of internal noise Ω^η is set to be $\Omega^\eta = \omega_\eta^2\, diag([1\,0\,0\,0\,0\,1\,0\,0\,0\,0])$, which means that the internal noise only affects the position update. Effect of assigning non-zero values for these zero terms will be tested in the sensitivity analysis.

As the start and the target position are known to subjects almost deterministically, we put the initial state covariance Σ_1 to be 0. Effect of non-zero initial positional uncertainty will be tested in the sensitivity analysis. The matrix for the signal dependent noise F_t is modelled as

$$F_t = \alpha \begin{bmatrix} \varepsilon_t^1 & \varepsilon_t^2 \\ \varepsilon_t^3 & \varepsilon_t^4 \end{bmatrix},$$

where α is a constant noise amplification factor ε_t^i's are random variables each of which follows a unit normal distribution. In theory, each element should have its own amplification factor, but we used one number for simplicity. Similar to the case of signal-independent noises, effects of having individual factors will be tested in the sensitivity analysis. BF_t is later decomposed into $\sum_{i=1}^4 \varepsilon_t^i C_i$ where each C_i has only one non-zero element α. Note that, since F_t has off-diagonal terms, the signal-dependent noise is coupled.

The state-dependent observation noise terms g_t, a, d are modelled as follows:

$$g_t = \begin{bmatrix} \beta\epsilon_t^1 \\ 0 \\ 0 \\ \beta\epsilon_t^2 \\ 0 \\ 0 \end{bmatrix}, \ d^\top = \begin{bmatrix} 0 & -cos(\theta) & 0 & 0 & 0 & 0 & -sin(\theta) & 0 & 0 & 0 \end{bmatrix},$$

which defines our experimental situation when the velocity in the visibility modulation direction (θ) affects the positional sensing. Note that a specifies the threshold above which the cursor becomes fully detectable. This state-dependent term is later decomposed into $\sum_{i=1}^{2} \epsilon_t^i(a_i + D_i x_t)$, where $a_1 = \beta a e_1$ and $a_2 = \beta a e_4$ (remember we defined e_m to be a standard basis vector with 1 at the m-th entry and 0 elsewhere). D_1 is a 6×10 matrices that has $D_{1,2} = -\kappa\beta cos(\theta)$, $D_{1,7} = -\kappa\beta sin(\theta)$ and zero elsewhere. D_2 is a 6×10 matrices that has $D_{4,2} = -\kappa\beta cos(\theta)$, $D_{4,7} = -\kappa\beta sin(\theta)$ and zero elsewhere. Note that, except when $\theta = 0, \pi/2$, this has two non-zero elements and therefore doesn't fit the assumption in Section 5, but can be transformed to a single non-zero element matrice if the coordinate frame is properly rotated so that one of the coordinate axis aligns to θ. Again, effects of having additional non-zero terms in g_t, such as an effect of state-dependent noise in the velocity sensing, will be tested in the sensitivity analysis.

6.1 Parameter selection

The system has the following eight free parameters to be selected: $[\tau,\ r, \alpha,\ w_v,\ w_f,\ \omega_\eta,\ \beta,\ a]$. For a movement of time step $\Delta \times N$-steps , we set $\tau = 0.04$, $r = {}^{10^{-6}}/_{(N-1)}$, $\alpha = 0.7^{\Delta}/_{\tau}$, $w_v = 0.3$, $w_f = 0.1$, $\omega_\eta = 0.005$, $\beta = 0.1$, and $a = 0.2$. Except unitless parameters, all values are based on MKS units. For sensitivity analysis, we add sixteen more parameters that were assumed to be zero (e.g., signal- or state-independent noise variances) or to be equal to the others (e.g. signal-dependent noise) in our simulation.

Additional parameters added are as follows:

1. $\xi_t = diag([\xi_t^p \ \ \xi_t^v \ \ \xi_t^f \ \ \xi_t^g \ \ 0 \ \ \xi_t^p \ \ \xi_t^v \ \ \xi_t^f \ \ \xi_t^g \ \ 0])^2$: 4 parameters
2. $\omega_t = diag([\omega_t^p \ \ \omega_t^v \ \ \omega_t^f \ \ \omega_t^p \ \ \omega_t^v \ \ \omega_t^f])^2$: 3 parameters
3. $\eta_t = diag([\eta_t^p \ \ \eta_t^v \ \ \eta_t^f \ \ \eta_t^g \ \ 0 \ \ \eta_t^p \ \ \eta_t^v \ \ \eta_t^f \ \ \eta_t^g \ \ 0])^2$ instead of $\eta_t^p \neq 0$ only: additional 3 parameters
4. $\Sigma_x^1 = diag([\sigma^p \ \ 0 \ \ \cdots \ \ 0])$: 1 parameter
5. $\alpha_{11},\ \alpha_{12},\ \alpha_{21},\ \alpha_{22}$ instead of α: 3 additional parameters
6. $a^1,\ a^2$ instead of a: 1 additional parameter
7. $\beta^1,\ \beta^2$ instead of β: 1 additional parameter.

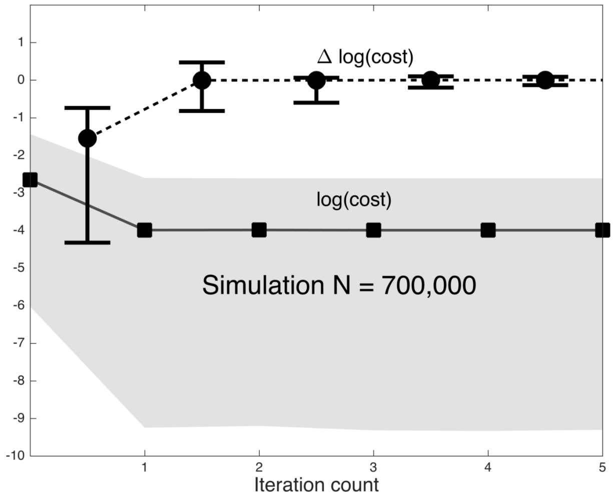

Figure S1: Cost per iteration. Total 700,000 simulations for all reaching directions in experiment 1. Total expected costs up to five iterations are summarized as the mean (filled squares) and the range (grey polygon) in a logarithmic scale. Changes in cost are summarized as the mean (filled circles) and the range (vertical lines), where a negative value indicates a decrease in cost.

By 5-7, F_t of the signal-dependent noise is now represented as

$$F_t = \begin{bmatrix} \alpha_{11}\varepsilon_t^1 & \alpha_{12}\varepsilon_t^2 \\ \alpha_{21}\varepsilon_t^3 & \alpha_{22}\varepsilon_t^4 \end{bmatrix},$$

and the state-dependent noise has effects in observation of the position and the velocity of the cursor:

$$g_t^1(a^1 + \kappa d^\top x_t) + g_t^2(a^2 + \kappa d^\top x_t),$$

where

$$g_t^1 = \left[\beta^1\epsilon_t^1, 0, 0, \beta^1\epsilon_t^2, 0, 0,\right]^\top, g_t^2 = \left[0, \beta^2\epsilon_t^3, 0, 0, \beta^2\epsilon_t^4, 0,\right]^\top$$

6.2 Convergence analysis

Figure S1 shows a summary of the cost per iteration for all 700,000 simulation runs (100,000 × 7 directions from 0 to 180 degrees) during the sensitivity analysis described in the main manuscript. We calculated the average, minimum and maximum costs for each iteration (up to fifth) in order to examine whether the suggested method can robustly reduce the total cost through iterations. The result suggests that the iterative method stably reduces the total cost over the iterations.

Reference

[1] Todorov E. Stochastic optimal control and estimation methods adapted to the noise characteristics of the sensorimotor system, Neural Comput; 2005;17(5);1084-108.
